# Vaccination with a non-human random sequence amyloid oligomer mimic results in improved cognitive function and reduced plaque deposition and micro hemorrhage in Tg2576 mice

**DOI:** 10.1186/1750-1326-7-37

**Published:** 2012-08-06

**Authors:** Suhail Rasool, Ricardo Albay, Hilda Martinez-Coria, Leonid Breydo, Jessica Wu, Saskia Milton, Sunit Misra, Andy Tran, Anna Pensalfini, Frank Laferla, Rakez Kayed, Charles G Glabe

**Affiliations:** 1 Department of Molecular Biology and Biochemistry, University of California, Irvine, CA 92697, USA; 2 Current affiliation: Department of Physiology and Neurosciences New York University School of Medicine New York, New York, NY 10016, USA; 3 Department of Neurology and Institute of Memory Impairment and Neurological disorders, University of California, Irvine, CA 92697, USA; 4 The George P. and Cynthia Woods Mitchell Center for Neurodegenerative Diseases, Department of Neurology, University of Texas Medical Branch, Galveston, TX 77555-1045, USA; 5 Departments of Neuroscience and Cell Biology, The University of Texas Medical Branch, Galveston, TX 77555-1045, USA

## Abstract

**Background:**

It is well established that vaccination of humans and transgenic animals against fibrillar Aβ prevents amyloid accumulation in plaques and preserves cognitive function in transgenic mouse models. However, autoimmune side effects have halted the development of vaccines based on full length human Aβ. Further development of an effective vaccine depends on overcoming these side effects while maintaining an effective immune response.

**Results:**

We have previously reported that the immune response to amyloid oligomers is largely directed against generic epitopes that are common to amyloid oligomers of many different proteins and independent of a specific amino acid sequence. Here we have examined whether we can exploit this generic immune response to develop a vaccine that targets amyloid oligomers using a non-human random sequence amyloid oligomer. In order to study the effect of vaccination against generic oligomer epitopes, a random sequence oligomer (3A) was selected as it forms oligomers that react with the oligomer specific A11 antibody. Oligomer mimics from 3A peptide, Aβ, islet amyloid polypeptide (IAPP), and Aβ fibrils were used to vaccinate Tg2576 mice, which develop a progressive accumulation of plaques and cognitive impairment. Vaccination with the 3A random sequence antigen was just as effective as vaccination with the other antigens in improving cognitive function and reducing total plaque load (Aβ burden) in the Tg2576 mouse brains, but was associated with a much lower incidence of micro hemorrhage than Aβ antigens.

**Conclusion:**

These results shows that the amyloid Aβ sequence is not necessary to produce a protective immune response that specifically targets generic amyloid oligomers. Using a non-human, random sequence antigen may facilitate the development of a vaccine that avoids autoimmune side effects.

## Background

Alzheimer's disease (AD) is associated with progressive cognitive decline, neuronal loss and the accumulation of senile plaques and neurofibrillary tangles in affected regions of the brain [1]. The original amyloid cascade hypothesis [2,3] proposed that the accumulation of amyloid plaques was the principal factor in AD pathogenesis. However recent studies indicate that small soluble Aβ aggregates or oligomers may represent the primary pathogenic entities [4-9]. One of the first tests of the therapeutic value of preventing the accumulation of Aβ or facilitating its clearance came from studies by ELAN, who immunized transgenic mouse models of AD with Aβ42 fibrils (fAβ42) [10]. Immunization with fAβ42 (AN1792) results in a nearly complete absence of Aβ plaque deposits, both in mice that were vaccinated prior to the onset of amyloid deposition and in animals that were vaccinated after amyloid deposition was well underway. However, human clinical trials were halted due to a high incidence of meningioencephalitis that is presumably due to an auto inflammatory reaction to immunization with human Aβ. Overcoming the auto inflammatory side effects while maintaining an effective immune response is a hurdle that must be overcome for the development of a human vaccine for AD [11].

We have previously shown that vaccination of transgenic mouse models of amyloid deposition with an Aβ oligomer mimic antigen is just as effective in reducing amyloid deposition and preventing cognitive dysfunction as vaccination with human fAβ42 [12]. The immune response to this oligomer mimic antigen results in the production of antibodies that recognize generic epitopes in prefibrillar oligomers independent of the precise amino acid sequence. Indeed, vaccination of rabbits with islet amyloid polypeptide (IAPP) oligomer mimics results in the production of an immune serum that specifically recognizes a variety of prefibrillar amyloid oligomers but not fibrils and is indistinguishable from the serum produced in response to vaccination with Aβ oligomer mimics [13]. This indicates that vaccination with the prefibrillar oligomer (PFO) conformation of other peptides gives rise to antibodies that recognize Aβ PFOs, suggesting that vaccination with a non-human random peptide sequence that also forms PFOs would also give rise to this generic PFO specific immune response. Recently, it was reported that vaccination of Tg mice with a 13 residue random sequence peptide derived from the carboxyl terminus of pABri (polymerized British amyloidosis peptide) related peptide reduces amyloid pathology and preserves cognitive function in APP/PS1 transgenic mice [14]. Here we report that vaccination with a random sequence PFO antigen gives rise to a generic PFO specific immune response that recognizes Aβ oligomers and that vaccination of Tg mice with this antigen is as effective in reducing amyloid deposition and preserving cognitive function. The use a non-human amyloid oligomer as a vaccine may overcome potential problems with auto inflammatory side effects observed with human Aβ.

## Results

### Identification of a random peptide sequence that forms A11 positive prefibrillar oligomers

**Figure 1:**
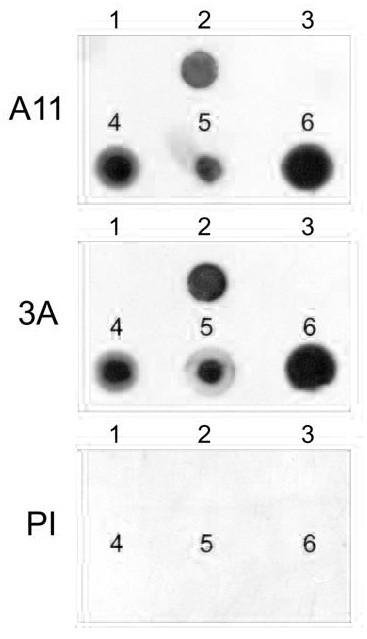
**Immune response to 3A oligomer mimics.** Serum from A11 vaccinated rabbits and 3A was used to probe dot blots containing Aβ monomer (1), Aβ prefibrillar oligomers (2), Aβ fibrils (3), calcitonin prefibrillar oligomers (4), IgG light chain prefibrillar oligomers (5) and synthetic KK(Q40)KK prefibrillar oligomers (6). It was observed that serum from 3A vaccinated rabbits reacted with all prefibrillar oligomer samples like A11 serum, but not Aβ monomer or Aβ fibrils. There was no immunoreactivity when using pre-immune serum.

We synthesized a series of 20 random sequence peptides from a restricted set of 8 amino acids and tested their ability to form prefibrillar oligomers by dot blot analysis with A11. Of these peptides, only 10 were sufficiently soluble to work with. One of these sequences, 3A, formed oligomers that react strongly with A11. We prepared 3A oligomer mimetic antigen by coupling 3A to colloidal gold particles and vaccinated rabbits with this immunogen. Serum from 3A vaccinated rabbits and A11 was used to probe dot blots containing Aβ monomer, Aβ prefibrillar oligomers, Aβ fibrils, calcitonin prefibrillar oligomers, IgG light chain prefibrillar oligomers and synthetic KK(Q40)KK prefibrillar oligomers. Like A11, which is raised against Aβ oligomer mimic antigen, serum from 3A vaccinated rabbits reacted with all prefibrillar oligomer samples, including Aβ, but not Aβ monomer or Aβ fibrils (Figure 1). No immunoreactivity was observed for preimmune serum from the rabbit immunized with 3A oligomers. These results demonstrate that vaccination with a non-human random peptide sequence oligomer mimic give rise to an immune response that recognizes many different types of prefibrillar oligomers, including Aβ and are consistent with our previous observations that serum from rabbits vaccinated with IAPP oligomer mimics is indistinguishable from A11 [13].

### Immune response in Tg2576 mice vaccinated with prefibrillar oligomer mimics and fibrillar Aβ

**Figure 2:**
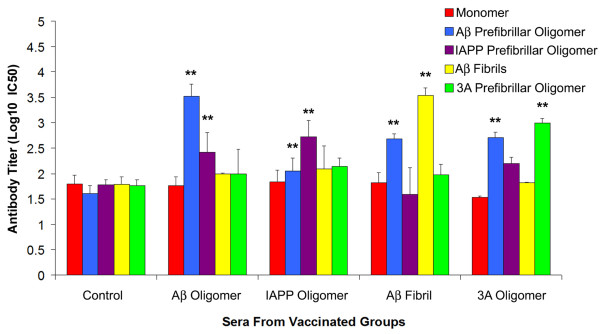
**Characterization of the immune response in vaccinated Tg2576 mice.** Antibody titers against Aβ monomer, Aβ prefibrillar oligomer, IAPP prefibrillar oligomer, Aβ fibril and 3A prefibrillar oligomer were determined for all the vaccinated groups by ELISA. Each vaccinated group showed significant immune response against its respective antigen as compared to the control vaccinated group. Values represent the average titer (mean ± SD) from the sera of 6 individual mice in each group (**p < 0.01).

To evaluate the therapeutic effectiveness of the 3A antigen, we immunized Tg2576 mice with 3A, Aβ, and IAPP oligomer mimetics and Aβ fibrils. Mice were immunized at monthly intervals beginning at 3 months up to 14 months. We performed ELISA assays to analyze the reactivity of the sera obtained from all groups of immunized mice against monomeric Aβ, Aβ PFOs, IAPP PFOs, 3A PFOs and Aβ fibrils (Figure 2). For all vaccinated groups, the highest reactivity is observed for the antigen they were immunized with. In addition, generic reactivity for prefibrillar oligomers samples is observed in the groups immunized with prefibrillar oligomer mimics. The immune response to the 3A peptide oligomer vaccine was specific for prefibrillar oligomers and did not display significant reactivity to monomeric or fibrillar Aβ (Figure 2). In contrast, the serum from Aβ fibrillar vaccinated mice shows immunoreactivity with Aβ fibril and Aβ PFOs (Figure 2). It shows that immunization with Aβ fibrils produced antibodies that reacted primarily with their own antigen and displayed some cross-reactivity with Aβ PFOs. Cross-reactivity could be due to small quantities of fibril-like structures in the PFO sample or small amounts of antibodies against PFOs in the serum. The titer of each vaccinated-group is expressed as the log10 of the IC50 values to normalize the distribution and statistical analysis on the transformed values has been done accordingly. As a result all the vaccinated groups, including IAPP oligomer, showed a significant immune response not only against their own antigen, but also against oligomers as compared to non-vaccinated mice. Control vaccinated mice displayed very low titers (<50) towards all antigens. None of the mice developed significant quantities of antibodies that react with Aβ40 monomer. These results indicate that the antibodies developed by the immunized mice in response to both Aβ fibrils and oligomers are predominantly conformation-dependent, aggregation specific and recognize generic epitopes that are independent of the precise amino acid sequence.

### Vaccination with 3A oligomer mimics improves cognitive performance

**Figure 3:**
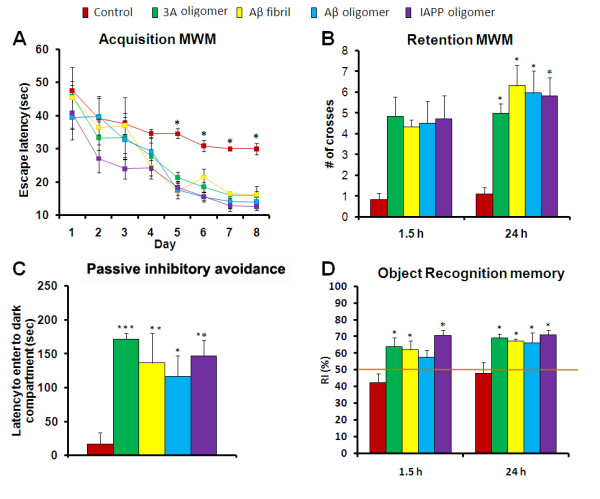
**Vaccination with 3A oligomer improves spatial cognitive deficits in Tg2576 mice. A**. 3A peptide-vaccinated and other vaccination groups of Tg2576 mice were evaluated using a spatial reference paradigm and compared with control treated mice vaccinated with colloidal gold and adjuvant. Mice were trained on the spatial reference version of the Morris water maze task after treatment. Besides 3A oligomer- also Aβ oligomer-, IAPP oligomer- and Aβ fibril-vaccinated mice also showed significant improvement (***p < 0.001) as compared to control vaccinated mice (Figure 1A). **B**. In probe trial during 1.5 hr and 24 hr testing, all vaccinated Tg2576 mice also display significantly shorter latencies than control vaccinated Tg2576 mice (ANOVA, ***p < 0.001, **p < 0.01 and *p < 0.05). **C**. Vaccination prevents contextual fear memory deficits in a mainly amygdala-dependent task. Mice were tested for retention of memory for fear-associated environments 24 hrs after training. Mice were taken out after 180 s if they did not cross over. At 24 hrs all the vaccinated mice showed significant improvement (***p < 0.001 for 3A vaccinated, **p < 0.01 for Aβ fibril, Aβ oligomer and IAPP oligomer vaccinated mice). **D**. Context dependent object recognition testing reveals that control vaccinated Tg2576 mice are impaired, spending an equivalent amount of time exploring both objects. At 14 months all the vaccinated mice showed improvement as compared to control vaccinated (*p < 0.05).

We evaluated the reference memory of Tg*2576* mice at the age of 14 months in a Morris water maze test. At the age of 14 months, mice were trained on the spatial reference version of the Morris water maze task after 11 months of vaccination. 3A peptide, Aβ oligomer, Aβ fibril and IAPP oligomer vaccinated mice all demonstrate a significant improvement (***p < 0.001) in behavioral acquisition and retention in Morris Water Maze (Figure 3A). At 90 minutes and 24 h in probe trials with the platform removed, mice immunized with Aβ oligomer, IAPP oligomer, 3A oligomer mimics and Aβ fibrils show significant improvement (***p < 0.001) in memory retention on the latency to cross the platform location and the number of platform crosses during 90 minute and 24 h tests as compared to controls (Figure 3B). Contextual learning and memory was evaluated using the passive inhibitory avoidance task (Figure 3C). Passive avoidance memory retention (mean ± S.E.M.) as measured by the ability of mice to remember an electrical shock after 24 hrs. Passive-avoidance tests were carried out in Tg*2576* mice at 14 months of age. In the first acquisition trial of the learning stage, all mice (14 months old) entered the dark compartment immediately after being placed in the illuminated compartment. In retention trials the step-through latency of the immunized mice with Aβ oligomer, IAPP oligomers and 3A oligomer mimics was significantly increased (*p < 0.05 for Aβ oligomer and ***p < 0.001 for IAPP and 3A peptide, ) as compared to that of the control immunized group (Figure 3C). We evaluated visual recognition memory at the ages of 14 months in a novel object recognition test (Figure 3D). This test is believed to be primarily dependent on cortex. The time that the mice explored the novel object versus the familiar object is called the recognition index (RI). 50% RI means that mice are not specifically interested in either of the objects. At 14 months, Tg2576 mice immunized with 3A oligomer mimics vaccinated mice shows statistically significant improvement in novel object recognition memory at both 1.5 hrs and 24 hrs (3D), to control vaccinated mice (*p < 0.05).

### Immunization with 3A oligomer mimics reduces **Aβ** plaque accumulation in Tg2576 mice

**Figure 4:**
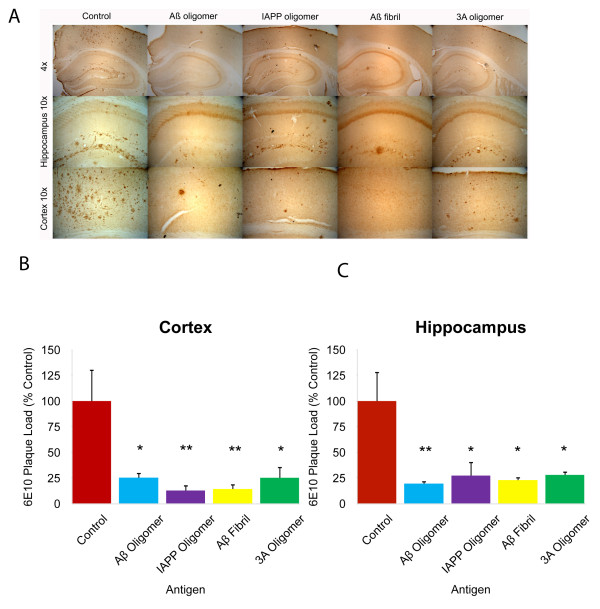
**Immunization initiated at 3 months of age in Tg2576 animals decreases total amyloid deposits. A**. Representative photomicrographs of sections from brains (hippocampus and cortex) of 14 months Tg2576 (Control, Aβ oligomer, IAPP oligomer, 3A peptide and Aβ fibril) that have been vaccinated as described in materials & methods from 3 to 14 months of age immunostained with 6E10 (which reacts with the human amyloid peptide). Scale bar: 100 microns **B**. Quantitation of plaque load. Image analysis (% Field area) of Aβ (6E10 antibody) immunoreactivity in hippocampus and cortex of immunized animals at 3–14 months is expressed as % of untreated control mice. The mean value for each animal was determined as the average of 2 sections (except untreated control which is 1 section per animal) using 4–8 images per section (most to all of the area of the section was analyzed). Bars represent group mean ± SEM of n mice per group: Control n = 8, Aβ oligomer n = 8, IAPP oligomer n = 8, 3A oligomer mimic n = 8 and Aβ fibril n = 6, *p < 0.05 and **p < 0.01 by ANOVA (**B**).

Vaccination against fibrillar Aβ is known to reduce plaque deposition in transgenic animals [10]. Amyloid deposition in 14 months Tg*2576* mice was assessed using the human Aβ specific monoclonal antibody, 6E10. We compared the effectiveness of the immunization on amyloid deposition by quantifying the amount of anti- Aβ (6E10) immunoreactive material. Figures 4A show representative photomicrographs from the hippocampus and cortex of control and immunized animals. Image analysis of sections from multiple animals demonstrated that Aβ deposits in Aβ oligomer, IAPP oligomer, 3A oligomers mimics and Aβ fibril immunized mice were decreased significantly as compared to control vaccinated group. (Figure 4B) shows that all the immunized groups displayed a significant reduction in Aβ plaque load in both hippocampus and cortex as compared to the respective controls (*p < 0.05, **p < 0.01).

### Immunization with 3A oligomer mimics reduces soluble and insoluble levels Aβ40 and Aβ42

**Figure 5:**
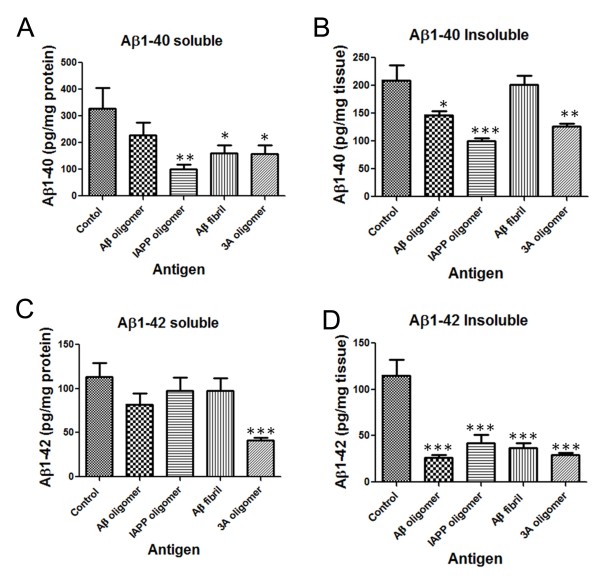
**Vaccination with 3A oligomer mimic strongly reduces levels of A**β**40 and A**β**42 in brain.** Following 3–14 months of treatment, levels of soluble and insoluble levels Aβ40 and Aβ42 on homogenates of the whole brain hemisphere of Tg2576 mice were determined by ELISA. 3A peptide treatment waswas associated with significantly lower levels of both soluble and insoluble Aβ40 and Aβ42. The insuluble levels were represented in pg/mg of tissue. Error bars represent standard errors of the mean (n = 8). *p < 0.05 for soluble levels of Aβ40, *p < 0.01 for soluble and insoluble levels of Aβ42, by one-way ANOVA test.

To evaluate whether vaccination with 3A peptide oligomer not only reduced plaque load but also affected cerebral total Aβ levels, brain homogenates were processed to prepare insoluble amyloid deposits*.* Subsequently, Aβ levels were quantified using a high-sensitivity sandwich ELISA measuring Aβ40 and Aβ42 separately as described in methods. Consistent with the reduced plaque by 3A oligomer mimics, we observed a significant reduction in soluble and insoluble levels of Aβ40 and Aβ42 (Figure 5). In Aβ fibril vaccinated mice, the insoluble levels of Aβ40 were not significantly decreased as compared to other groups, where as insoluble Aβ42 levels were significantly decreased. Immunization with the 3A peptide oligomer mimic antigen induces an immune response that significantly reduced insoluble levels of cerebral Aβ40 and Aβ42.

### Immunization with 3A Oligomer mimics reduces microglial activation

**Figure 6:**
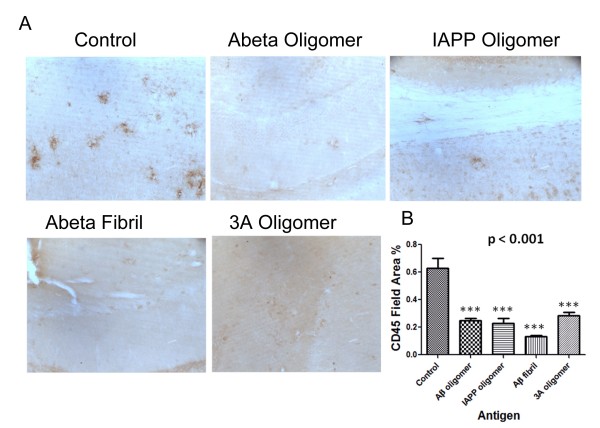
**Vaccination of animals at 3–14 months with antigens decreases anti CD45 reactivity. A**. Following vaccination with 3A oligomer mimics significantly reduces the activated microglia. Representative photomicrographs of CD45 (brown) staining of brain sections from 14 months Tg2576 control, or vaccinated with Aβ oligomer, Aβ fibril, IAPP oligomer and 3A peptide. Scale bar 50 microns. **B**. Quantitation of CD45 immunoreactivity in cortex and hippocampus of animals immunized at 3–14 months. Mean of each animal is the average of two sections in which most to all the area of study was analyzed (4–8 images per section) Bars represent mean ± SD of n mice per group. *p < 0.02; CD45: Control n = 4, Aβ oligomer vaccinated n = 4, Aβ fibril vaccinated n = 4, IAPP oligomer vaccinated n = 4, 3A oligomer mimics vaccinated n = 4 *** p < 0.001.

Microglia play a major role in regulating homeostasis in the brain and have the ability to activate phagocytose, secrete cytokines, and to present antigens to T cells depending on their stimulatory environment [15]. However, some of these same neuroprotective functions are detrimental if dysregulated [16]. One of the main characteristics accompanying accumulation of Aβ plaques in both human Alzheimer’s brain and transgenic mouse models of AD is an enhanced neuroinflammatory response characterized by activation of microglia. It has been previously reported that immunization with Aβ reduces the microglial activation [12]. We assessed the effect of immunization by 3A oligomer mimics on microglial reactivity as measured by CD45 immunohistochemistry (Figure 6A). All the immunized groups displayed a significant decrease in CD45 immunostaining relative to the respective controls (***p < 0.001) (Figure 6B).

### GFAP reactivity is decreased in 3A oligomer mimics vaccinated mice

**Figure 7:**
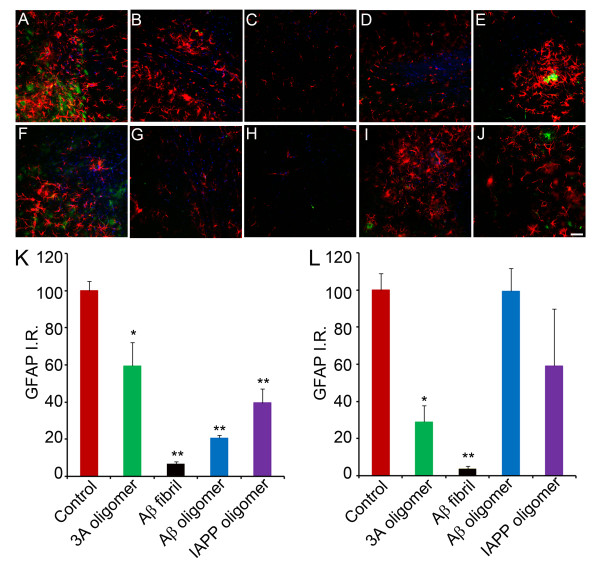
**Activated astrocytes surrounding Aβ plaques are decreased in Tg2576 mice following vaccination.** Astrocyte reactivity (GFAP, red) preferentially associates with Aβplaques (6E10, green) and is reduced in the hippocampus (**A**–**E)** and cortex (**F**–**J**) of Tg2576 mice following immunization with PBS control (**A**, **F**), 3A oligomer mimics (**B**, **G**), Aβ fibrils (**C**, **H**), Aβ oligomers (**D**, **I**), and IAPP oligomers (**E**, **J**). Cell nuclei were stained with DAPI (blue). Scale bar = 100 μm. Quantifications of GFAP immunoreactivity in the hippocampus (**K**) and cortex (**L**) demonstrate significant astrocytosis reduction in mice vaccinated with 3A and Aβ fibrils. Error bars represent standard errors of the mean (n = 3). *p < 0.05, ** p < 0.001 by one-way ANOVA test.

Activation of astrocytes is another characteristic feature of amyloid deposition. It has been observed that activated astrocytes are reduced in the same proportion as the amyloid by immunization with Aβ [12]. To assess the involvement of activated astrocytes following vaccination, immunohistochemical analysis of GFAP, a marker for astroglia was performed. Quantitative analysis showed that there was a significant reduction in GFAP immunoreactivity. 3A peptide oligomer mimic immunized mice display a significant reduction of activated astrocytes in the hippocampus and cortex (Figure 7A-J). The quantification was statistically significant as compared to controls (*p < 0.05). Activated astrocytes were also significantly decreased in hippocampus of mice vaccinated with Aβ oligomer and IAPP oligomer, however in cortex there was no significant decrease. The explanation for this difference may be that in Aβ oligomer and IAPP oligomer vaccinated mice where we observed a decrease in cortical plaques, there is GFAP immunoreactivity that is not associated with plaques.

### Microhemorrhage is reduced in 3A oligomermimics and IAPP oligomer vaccinated mice

**Figure 8:**
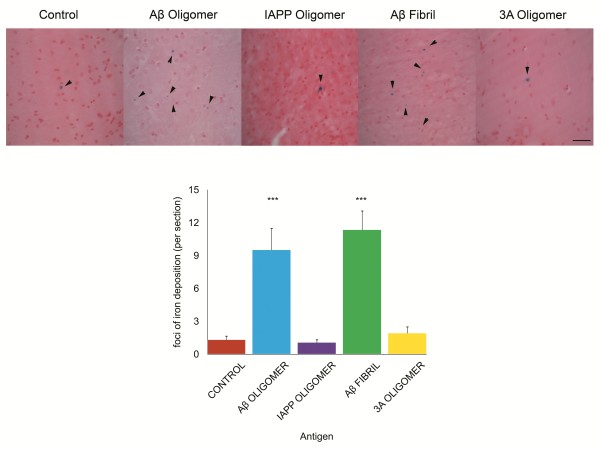
**No increase in incidence of microhemorrhage in 3A and IAPP oligomer mimic vaccinated mice. A**. Representative images of Prussian blue stained sections. Arrowheads point to typical stage 1 microhemorrhages. Scale bar: 25 microns. **B**. The incidence of microhemorage was counted after staining for hemosiderin. Both the Aß oligomer mimic and Aß fibril vaccinated groups display a much higher incidence of microhemorrhage than the control, 3A or IAPP oligomer vaccinated groups. The mean counts from 3 different sections of each animal is shown +/− the standard error of the mean (n = 8). ***P < 0.001. The 3A oligomer mimics and IAPP vaccinated group.

An increased incidence of micro hemorrhage has been observed in both active and passive Aß immunotherapy [17]. We therefore investigated the incidence of micro hemorrhage in the vaccinated and control animals. The method we used categorizes micro hemorrhages in 4 different stages of increasing size and vessel involvement [18]. The number of micro hemorrhages per section is reported in Table 1 for each stage. As previously reported, most of the micro hemorrhages observed in Aß vaccinated Tg2576 animals were stage 1, consisting of 1 to 5 grains of iron or small vessel involvement and are shown in Figure 8A. We found that the 3A and IAPP vaccinated mice showed a striking reduction in parenchymal iron deposition consistent with a lower incidence of micro hemorrhages compared to the Aβ oligomer and Aβ fibril vaccinated groups (***P < 0.001) (Figure 8B). The 3A and IAPP vaccinated groups were not statistically different from the control vaccinated group. Stage 2 micro hemorrhages, consisting of multiple grains of iron and micro vessel involvement were also significantly elevated in the Aß fibril and Aß oligomer vaccinated groups (Table 1, P < 0.001, P < 0.05 respectively). Stage 3 micro hemorrhages, consisting of several positive micro vessels in 1 area, were significantly elevated only in the Aß fibril vaccinated group. Stage 4 micro hemorrhages with large blood vessel involvement were rarely observed in the Aß vaccinated groups and none were observed in the control, 3A and IAPP vaccinated groups although this difference did not reach statistical significance. In Aß vaccinated fibril and oligomer groups, micro hemorrhages were observed in the somatosensory and auditory cortices, the CA1, CA3 and dente gyrus of the hippocampus, lateral and ventral hypothalamic areas, and the ventral posterior medial thalamic nucleus. No preferential localization of micro hemorrhages in a specific brain area was observed for any groups. These results indicate that the non-Aβ antigens may have a better safety profile with respect to micro hemorrhage than Aβ containing antigens.

**Table 1:** Microhemorrhage foci observed per section

**Group**	**Stage 1**	**Stage 2**	**Stage 3**	**Stage 4**
Control	0.9	0.5	0	0
3A	1.7	0.2	0	0
IAPP	1.0	0.1	0	0
Aß oligomer	6.9***	1.6*	0.6	0.4
Aß fibril	7.6***	2.5***	0.9***	0.3

Statistical significance in comparison to control, 3A and IAPP groups:

*** P < 0.001.

* P < 0.05.

## Discussion

Anti-Aβ immunotherapy is currently one of the leading strategies for AD therapeutic development with several vaccines in human clinical trial [2,19]. Numerous pre-clinical studies with mouse models of AD have demonstrated the ability of Aβ vaccination to prevent amyloid deposition in the brain. Thus, active immunization with Aβ [10,20], as well as passive immunization [15-17,19-27] resulted in a reduction of the amyloid burden in the brain. Importantly, active immunization prevented cognitive decline [28,29] and passive immunizations were shown to reverse the memory loss in aged Tg2576 mice [30]. While these and other studies did not report any adverse events in AD transgenic mouse models immunized with fibrillar Aβ42, data from the AN1792 vaccine trial reported meningoencephalitis in 6% of the patients. These adverse reactions to Aβ42-immunotherapy appear not to be due to the humoral antibody response, but rather to the cell-mediated autoimmune response which induced a Th1 type immune response in patients that received the AN1792 vaccine [31-37]. Thus, the goal of this pre-clinical study was to investigate the effectiveness of a non human random peptide (oligomer mimic), that induces a Th2-type humoral immune response specific to oligomeric Aβ peptide, but would not be expected to cause Th1 auto inflammatory side effects because the antigen is non-human.

The data presented here indicate that immunization with non-human 3A amyloid random sequence oligomer produces an efficacious immune response in a murine model of AD that is comparable to that of mice immunized with fibrillar Aβ42 and Aβ40 oligomer mimics. We tested escape latency, number of platform crosses in the Morris water maze test (MWM) (which is related to hippocampus), novel object recognition (which is related to cortex) and inhibitory avoidance (which is related to amygdala). It was found that at 14 months, 3A oligomer mimic vaccinated mice have a significant improvement in cognitive function compared to controls. We examined the effect of immunization on the neuropathology in 14 months old mice, and showed a significant reduction in plaques in both cortex and hippocampal region immunized with 3A peptide. The ELISA data shows that the reduction in insoluble Aβ42 is modest (approximately 40%) and mostly absent for Aβ40 in the fibril vaccinated group, while we observed a more robust (approximately 80%) decrease in plaque burden as determined by 6E10 immunoreactivity by IHC. The explanation for this apparent discrepancy is not clear, but it may be due to the fact that the ELISA measures the amount Aß while the immunohistochemistry measures area of the plaque. This may be a reflection of a change of state of the amyloid in the fibril vaccinated group to a more compact or dense state rather than its removal.

These results are consistent with recent findings that vaccination against a random sequence peptide encoded by read through of a stop codon in the pABri mRNA improves cognition and reduces plaques in APP/PS1 mice [14]. Mice immunized with the ABri random peptide produced antibodies that recognize aggregated Aβ, reduced plaque deposition and improve cognition, similar to the results we report here. However, the immune response to this antigen is broader and includes antibodies that react with neurofibrillary tangles and plaques. Together with our results with the 3A random sequence antigen, these results provide strong support to the concept that targeting generic epitopes associated with Aβ aggregates is therapeutically effective. Small but significant levels of this type of antibodies exist in non-vaccinated humans and the levels of these antibodies are inversely correlated with the incidence of AD [38]. These conformation-specific, sequence-independent antibodies are the major antibodies against Aβ that exist in normal human plasma [38] and these antibodies may account for the reported effectiveness of IVIg in reducing the incidence of AD in humans [39]. IAPP oligomer and 3A oligomer mimics vaccinated mice showed a relatively low titer as compared to Aβ oligomer and Aβ fibril vaccinated groups, yet retaining their efficacy in preventing cognitive deficits and amyloid deposition. Increasing evidence suggests a role for amyloid oligomers as the toxic species responsible for disease progression and pathogenesis independently of the plaque load [14]. In this view, our results suggest that effective targeting of toxic species rather than the overall extent of immune response elicited may be relevant. The higher titer observed for either Aβ oligomer and Aβ fibril vaccinated groups may therefore reflect the recognition of a broader range of aggregated conformations.

There is increasing evidence that Aβ oligomers and fibrils are conformationally and structurally diverse [40-42], which raises the issue of whether the different conformers are differentially associated with pathogenesis [43]. Because monoclonal antibodies recognize these conformers in a mutually exclusive fashion [44,45], a single antibody may not be able to target all of the pathologically significant forms of Aβ. The polyclonal response to active immunization is broad, suggesting that it may be more beneficial in its ability to target more different conformers of Aβ than a single monoclonal [45]. A potential advantage of vaccination against conformation dependent epitopes is that the resulting antibodies are specific for aggregated forms of Aβ and do not react with Aβ monomer or APP [13,40]. This suggests that these antibodies may have a better pharmacological profile than antibodies that recognize APP, which is abundantly expressed or Aβ monomer which is a normal product of APP processing and may have normal roles that should not be interfered with. The lack of reactivity with normal human proteins would also be expected to provide a lower potential for autoimmune complications as well.

We also found that the non-Aβ antigens, 3A and IAPP were associated with a much lower incidence of micro hemorrhage than the Aβ oligomer and Aβ fibril groups. Since the conformation dependent immune response to Aβ oligomer and Aβ fibril antigens are distinct while the immune response to the oligomer mimic antigens is common [13,40], this suggests that the increased incidence of micro hemorrhage may be due to sequence specific antibodies that are common to the Aß containing antigens. This reduced incidence of micro hemorrhage may also contribute to a superior safety profile of the random sequence oligomer antigen, like 3A.

These results demonstrate that vaccination against generic amyloid oligomer epitopes is capable of attenuating cognitive impairment and producing a protective immune response without increasing the incidence of micro hemorrhage, suggesting that vaccination against a non-human amyloid oligomer epitope may be a safer therapeutic strategy for developing an effective vaccine that circumvents auto inflammatory immune complications.

## Materials and methods

### Preparation of random peptides

We synthesized a series of 8 random sequence 20mers using a restricted set of 8 amino acids, threonine, tyrosine, serine, histidine, isoleucine, valine, phenylalanine and leucine. The sequences of the 20mers were randomly drawn and checked against the human non-redundant protein sequence data base with BLAST to select for sequences that had the lowest homology with human protein sequences. The sequences were synthesized and tested for the ability to form prefibrillar amyloid oligomers using the A11 prefibrillar oligomer specific polyclonal antibody. One of the sequences (peptide 3A: TYLIHVHIITIYHISIYYIV) formed A11 positive oligomers in a time dependent fashion and was selected for further analysis.

### Oligomer mimic antigens

Aβ, IAPP and 3A peptide oligomer molecular mimic antigens were prepared by synthesizing carboxyl-terminal thiol derivatives and coupling them to colloidal gold particles as previously described [46].

### Preparation of Aβ prefibrillar oligomers and fibrils

Aβ40 peptides were lyophilized in 50% acetonitrile/ddH_2_O. Soluble oligomers were prepared by dissolving 1.0 mg of peptide in 400ul hexafluoroisopropanol (HFIP) for 10–20 min at room temperature. 100 μl of the resulting seedless solution was added to 900 μl milliQ H_2_O in a siliconized Eppendorf tube. The samples were then stirred at 500 RPM using a Teflon coated micro stir bar for 24–48 h at 22°C. The samples were centrifuged for 15 min. at 14,000 × G and the supernatant fraction (pH 2.8–3.5) was transferred to a new siliconized tube and subjected to a gentle stream of N_2_ for 5–10 min to evaporate the HFIP. Formation of oligomers were confirmed by atomic force microscopy (AFM), electron microscopy (EM) and size exclusion chromatography (SEC) as described [40]. Fibrils were formed by dissolving Aβ42 in 50% HFIP/ddH_2_O and stirred with closed caps for 7 days. Solution was stirred again for 2 days using open caps to evaporate the HFIP. Fibrils were sedimented, washed, and resuspended in PBS at 2 mg/ml.

### Immunization

New Zealand white rabbits were immunized with 3A oligomer mimics as previously described and the serum collected after significant titer against Aβ oligomers was detected [46]. Preimmune serum was collected from the same rabbits prior to immunization. We immunized young Tg2576 mice (3 months old) [5] with one of the following antigens: Aβ oligomer, IAPP oligomer, Aβ fibril, random peptide 3A oligomers or control with colloidal gold in PBS. The mice were equally distributed in each group of eight (4 males and 4 females). The antigens were mixed with incomplete Freund’s adjuvant (1:1,v/v) and immunization was done subcutaneously (100 μg/immunization) every month up to 14 months. For controls a mixture of PBS and colloidal gold was used.

### Behavioral studies

Three common tests for cognitive dysfunctions in AD model mice, the Morris Water Maze (MWM) [47,48] novel-object recognition [48,49], and passive inhibitory avoidance tests [10], were performed according to previous reports with minor modifications.

#### Morris water maze

Morris water maze is a special memory task related to hippocampus. The apparatus used for all water maze tasks was a circular aluminum tank (1.5 m diameter) painted white and filled with water maintained at 26°C–29°C. The maze was located in a room containing simple visual, extra-maze cues. To reduce stress, mice were placed on the platform in both the hidden and cued versions of the task for 15 sec. prior to the first training trial. Mice were trained to swim to a circular clear Plexiglas platform (14 cm diameter) submerged 1.5 cm beneath the surface of the water and invisible to the mice while swimming. The platform location was selected randomly at 14 months test, but was kept constant for each individual mouse throughout training. On each trial, the mouse was placed into the tank at one of four designated start points in a pseudorandom order. Mice were allowed 60 sec. to find the submerged platform. If a mouse failed to find the platform within 60 sec., it was manually guided to the platform and allowed to remain there for 15 sec. After this, each mouse was placed into a holding cage under a warming lamp for 30 s before beginning the next trial. To ensure that memory differences were not due to lack of task learning, mice were given four trials a day for as many days as were required to train the Tg2576 mice to reach the criterion (<20 sec.). To control for overtraining, probe trials were run for each group, both as soon as they reached group criterion and after all groups had reached criterion. We trained the Tg2576 mice for 8 days at 14 months. Retention of the spatial training was assessed 1.5 hr and again 24 hr after the last training trial. Both probe trials consisted of a 60 s free swim in the pool without the platform. Mice were monitored by a camera mounted in the ceiling directly above the pool to record the 1.5 hr and 24 hr test. The parameters measured during the probe trial included initial latency to cross the platform location, number of platform location crosses, and time spent in the quadrant *opposite* to the one containing the platform during training.

#### Novel object recognition

The object recognition task used is based on the spontaneous tendency of rodents to explore a novel object longer than a familiar one. On day 1, the mice were allowed to familiarize themselves with the empty open field for 5 minutes. On day 2, they were subjected to a 5-minute exploration session of two identical, symmetrically placed objects A. Ninety minutes and 24 hours later, the animals were subjected to a 3-minute retention session where they were exposed to one object A and to a novel object B (after 90 minutes) or object C (after 24 hours). The times of exploration were recorded, and an object recognition index (ORI) was calculated, such that ORI = (tn - tf)/(tn + tf), where tf and tn represent times of exploring the familiar and novel objects, respectively.

#### Passive inhibitory avoidance

Amygdala dependent task was evaluated using the passive inhibitory avoidance task [21], performed in the Gemini Avoidance System (San Diego Instruments, San Diego, CA). The training trial consisted of placing a mouse in the illuminated compartment of the device and recorded the latent time required for the mouse to enter the dark compartment (baseline latency). Upon entering the dark chamber, the door between the two compartments was closed and the animal was immediately given an electric shock to the feet (0.15 mA, 1 s). During the retention trials (conducted 1.5 h and 24 h after the training trial), the mouse was again placed in the illuminated compartment and the latency to enter the dark compartment was recorded. The retention trial was interrupted if the animal took more than 180 seconds to cross into the dark compartment.

### ELISA analysis of anti Aβ antibodies

ELISA assays were performed as previously described [40]. Briefly, 200 ng/100 μl of antigen (Aβ oligomer, IAPP oligomer, 3A oligomer mimic, Aβ fibril and Aβ monomer) was plated on ELISA wells and blocked with BSA. Serum samples were serially diluted to an end point of 1:100,000. The secondary antibody used for detection is peroxidase conjugated AffiniPure Goat Anti-mouse IgG (H + L) (Jackson ImmunoResearch). Titer was determined from the midpoint of the dilution curve (IC50).

### Tissue collection and immunohistochemistry

After mice were anesthetized with pentobarbital (150 mg/kg, IP), blood was collected by cardiac puncture, and mice were perfused transcardially with cold phosphate-buffered saline (PBS). Brain tissues were fixed overnight with 4% paraformaldehyde in PBS, pH 7.4 at 4°C and stored in PBS/0.02% sodium azide (NaN_3_) at 4°C until use. Fixed brain tissues were sectioned (40 μm) with a vibratome. Coronal sections were collected in PBS (containing 0.02% sodium azide) and stored at 4°C prior to staining. To stain for Aβ plaques, sections were immersed in 70% formic acid for 5 min. Endogenous peroxidase in tissue was blocked by treating with 3% H_2_O_2_ in PBS for 10 min at room temperature. Nonspecific background staining was blocked by 1 h incubation in 2% BSA, 0.3% Triton X-100 (TX) at room temperature. Tissues were incubated with primary antibodies (6E10, GFAP and CD45) overnight at 4°C, rinsed 3 times with PBS,0.1% TX, followed by biotinylated secondary antibodies (anti-rabbit, anti-mouse and anti-rat), detection with an ABC peroxidase kit, and visualization with a 3,3'-diaminobenzidine (DAB) substrate kit (Vector, Burlingame, CA). CD45 (Serotec, Raleigh, NC; 1:3000) staining was done as described previously [22]. Control experiments with primary or secondary antibody omitted resulted in negative staining.

### Immunohistochemical analysis

Coronal brain sections were double-immunostained for astrocytes and Aβ plaques. Sections were permeabilized in 0.1% Triton X-100 for 15 min at RT, blocked in 5% bovine albumin/PBS for 1 hour, and then probed with anti-mouse glial fibrillary acidic protein, GFAP (1:1000, Sigma), rabbit anti-fibril OC (1:4000) [13] for 24 h at RT. Fluorescent-conjugated secondary antibodies (anti-mouse, anti-rabbit Alexa, 1:400, Invitrogen Molecular Probes) were used for detection. Double immunofluorescent images were captured at a magnification of 10x and anayzed by the z-stack confocal microscopic system using Axio Vision (Zeiss).

### Image quantification

Immunostaining was observed under a Zeiss Axiovert-200 inverted microscope (Carl Zeiss, Thornwood, NY) and images were acquired with a Zeiss Axiocam high-resolution digital color camera (1300x1030 pixel) using Axiovision 4.1 or 4.6 software. The same software (Carl Zeiss) was used to analyze the digital images. Percent of immunopositive area (% Field Area) (immunopositive area/total image area × 100) was determined for all the markers studied by averaging images of the cortex, hippocampus and subiculum area from 2–3 sections per animal. Digital images were obtained using the same settings and the segmentation parameters constant within a range per given marker and experiment. The mean value of the% Field Area for each marker in each animal was averaged per genotype group with the number of animals per group indicated in Figure legends.

### Enzyme-Linked Immunosorbent Assay (ELISA) for soluble and insoluble Aβ

Soluble and insoluble Aβ fractions were isolated from whole brain homogenates using four step extraction protocol [24]. Frozen hemibrains were sequentially extracted. At each step, sonication in an appropriate buffer was followed by centrifugation at 100,000 x *g* for 1 hr at 4°C. The supernatant was then removed, and the pellet was sonicated in the next solution used in the sequential extraction process. For four-step extraction, sonication of the frozen brain (150 mg/ml wet weight) began in Tris-buffered saline (TBS) (20 mM Tris and 137 mM NaCl, pH 7.6), which contained protease inhibitors (Protease inhibitor cocktail from Sigma St. Louis USA). The next three sequential extraction steps used 1% Triton X-100 in TBS with protease inhibitors, 2% SDS in water with the same protease inhibitors, and 70% formic acid (FA) in water. Soluble fractions were loaded directly onto ELISA plates, whereas insoluble fractions were diluted 1:20 in a neutralization buffer (1 mol/L Tris base, 0.5 mol/L NaH2PO4) before loading. MaxiSorp immunoplates (Nunc, Rochester, NY) were coated with Mab 20.1 antibody (a kind gift from Dr. David Cribbs University of California Irvine) at a concentration of 25 μg/ml in coating buffer (0.1 M/L Na2CO3, pH 9.6) and blocked with 3% bovine serum albumin. Standard solutions for both Aβ40 and Aβ42 were made in the antigen capture buffer (20 mmol/L NaH2PO4, 2 mmol/EDTA, 0.4 M NaCl, 0.05% 3-[(3- cholamidopropyl) dimethylammonio] propanesulfonate, and 1% bovine serum albumin, pH 7.0) and loaded onto ELISA plates in duplicate. Samples were then loaded (also in duplicate) and incubated overnight at 4°C. Plates were then washed and probed with either horseradish peroxidase conjugated anti-Aβ40(C49) or anti-Aβ42(D32) overnight at 4°C. The chromogen was 3,3_,5,5 tetramethylbenzidine, and the reaction was stopped by 30% phosphoric acid. The plates were read at 450 nm using a plate reader (Molecular Dynamics, Sunnyvale, CA). The readings were then normalized to protein concentrations of the samples.

#### Micro hemorrhage analysis

Coronal brain sections were stained for hemosiderin as previously described [18]. 3 different sections were counted for each mouse and scored independently by two investigators as stage 1–4. Stage 1 is defined as “1 to 5 grains of iron or small micro vessel involvement”; stage 2 is “multiple grains of iron and micro vessel involvement”; Stage 3 is “several positive micro vessels in 1 area”; and Stage 4 is “large blood vessel involvement”. The data shown are the averages of the two independent countings and are presented as the mean +/− the standard error of the mean.

#### Statistical analysis

All statistical analysis were performed using GraphPad Prism 5 (GraphPad Software, San Diego, CA). For comparison between antigen vaccinated groups and control vaccinated group, one-way analysis of variance (ANOVA) followed by Dunnet post-test was performed. Probability values less than 0.05 were accepted as statistically significant.

## Competing interests

The authors declare no competing interests.

## Authors' contributions

SR prepared the antigens, immunized the Tg2576 mice and did the biochemical and immunological characterization. HM-C and AT did the cognitive function tests. LB and AP did the sera titration. JW did the activated astrocyte quantification. SM synthesized the peptides. RA performed the ELISA. SuM and RA conducted the micro hemorrhage analysis. RK characterized the 3A oligomer mimics immunoreactivity. FLaF analyzed the behavioral data. CG participated in concept, design, data analysis and manuscript preparation. All authors read and approved the final manuscript.
